# Response to ‘Refined analyses suggest that recombination is a minor source of genomic diversity in *Pseudomonas aeruginosa* chronic cystic fibrosis infections’ by Williams *et al.* (2016)

**DOI:** 10.1099/mgen.0.000054

**Published:** 2016-03-02

**Authors:** Sophie E. Darch, Alan McNally, Jukka Corander, Stephen P. Diggle

**Affiliations:** ^1^​ School of Life Sciences, University of Nottingham, Nottingham NG7 2RD, UK; ^2^​ Department of Molecular Biosciences, Institute of Cellular and Molecular Biology, Center for Infectious Disease, The University of Texas at Austin, Austin, TX 78712, USA; ^3^​ Pathogen Research Group, Nottingham Trent University, Nottingham, UK; ^4^​ Department of Mathematics and Statistics, University of Helsinki, Helsinki, Finland

The paper ‘Refined analyses suggest that recombination is a minor source of genomic diversity in *Pseudomonas aeruginosa* chronic cystic fibrosis infections’ by Williams *et al.* (2016) performs a new analysis on data from a recent paper of ours where we found that recombination plays a significant role in generating phenotypic and genomic diversity in *P. aeruginosa* populations taken from the lungs of cystic fibrosis patients (Darch *et al.*, 2015). They compare our dataset with a recent dataset of their own (Williams *et al.*, 2015) and conclude that whilst recombination exists, it has only a small effect on population heterogeneity when compared with *de novo* mutation.

The authors present an extremely well performed analysis of the two datasets and, furthermore, they also present a new tool for high-confidence SNP calling in studies containing such datasets. The analysis in Williams *et al.* (2016) is sound and is a valuable addition to the literature; however, this does not mean that their analysis approach and conclusions are right whilst those presented by us are wrong. If one compares the mapping results presented by Williams *et al.* (2016) with the mapping data presented in our study, there is actually a good degree of congruence, although the new approach developed by Williams *et al.* (2016) does appear to filter out SNPs not done so by smalt and SAMtools and this is an encouraging step forward.

Williams *et al.* (2016) suggest that the recombination inference of our study is inaccurate as detected by many of the recombination events occurring in what are seen to be repeat regions of the reference genome which map poorly. However, the recombination we detected used *de novo* assembled genomes. This decision was taken to allow for better detection of any novel acquisitions or large deletions in the dataset. We assembled our genomes using pagit, which works by taking a Velvet genome assembly, reordering the contigs according to a reference strain (in this case the LESB58 genome), and then using iCORN and image to iteratively map reads back against the genome assembly to close gaps, correct aberrant base calls and rearrange regions undergoing inversions. Thus, the regions identified as containing potential recombination events are entirely inferred by comparison of *de novo* assemblies. We also decided to use this approach to infer insertions and deletions as using variant calling pipelines to assign short indels with high levels of confidence can be problematic (O'Rawe *et al.*, 2013).

In our study, we were careful to exclude any potential recombination events occurring at the ends of contigs, and excluded several regions which were inferred due to low coverage areas and ambiguous N called regions. What we reported was a conservative inclusion of regions that had the highest confidence. The fact that we observed recombining regions strongly associated with particular phenotypes suggests that these regions may be robust, as one would expect the associations to be randomly distributed if they were errors, or indeed present in every genome assembly if the same regions are troublesome during the assembly process, but this was not the case.

There has been considerable debate over the merits of mapping and assembly approaches in microbial genomic studies (Bertels *et al.*, 2014). The argument for mapping is that phylogenetic inferences are far more accurate, and for assembly-based studies that the biological information available is far greater and less susceptible to removal of true variations. Therefore, we believe that the key point of this new study is not that one approach is right and the other wrong, it is that two different analyses can give rise to different biological outcomes and interpretations. As long read sequencing becomes more common place in microbial genomics via PacBio and Oxford Nanopore technologies, the hope is that assembly-based methods will allow microbiologists to generate data with unrivalled levels of accuracy and resolution. Combined with a strong collaborative ethos across research groups this should allow the microbiology community to fully address questions such as the levels of gene flow and variation between very closely related bacterial communities.
